# 4-Amino-*N*-(2,3-di­hydro-1,3-thia­zol-2-yl­idene)benzene­sulfonamide–2,4,6-tris­(pyr­idin-2-yl)-1,3,5-triazine (1/1)

**DOI:** 10.1107/S1600536814004838

**Published:** 2014-03-08

**Authors:** Hadi D. Arman, Trupta Kaulgud, Edward R. T. Tiekink

**Affiliations:** a Department of Chemistry, The University of Texas at San Antonio, One UTSA Circle, San Antonio, Texas 78249-0698, USA; b Department of Chemistry, University of Malaya, 50603 Kuala Lumpur, Malaysia

## Abstract

The sulfa­thia­zole mol­ecule in the title 1:1 co-crystal, C_9_H_9_N_3_O_2_S_2_·C_18_H_12_N_6_, adopts an approximate L-shape [dihedral angle between the five- and six-membered rings = 86.20 (9)°] and features an intra­molecular hypervalent S⋯O inter­action [2.8666 (15) Å]. Overall, the triazine mol­ecule has the shape of a disk as the pendant pyridine rings are relatively close to coplanar with the central ring [dihedral angles = 18.35 (9), 6.12 (9) and 4.67 (9)°]. In the crystal packing, a linear supra­molecular chain aligned along [01-1] is formed as a result of amino–pyridyl N—H⋯N hydrogen bonding with *syn*-disposed pyridyl mol­ecules of one triazine, and amine–pyridyl N—H⋯N hydrogen bonding with the third pydridyl ring of a second triazine mol­ecule. A three-dimensional architecture arises as the chains are connected by C—H⋯O inter­actions.

## Related literature   

For previous co-crystallization studies with sulfa­thia­zole, see: Arman *et al.* (2012 ▶). For the polymorphic 1:1 co-crystals of sulfa­thia­zole and pyridine, see: Drebushchak *et al.* (2006*a* ▶,*b* ▶). For hypervalent S⋯O inter­actions, see: O’Leary & Wallis (2007 ▶).
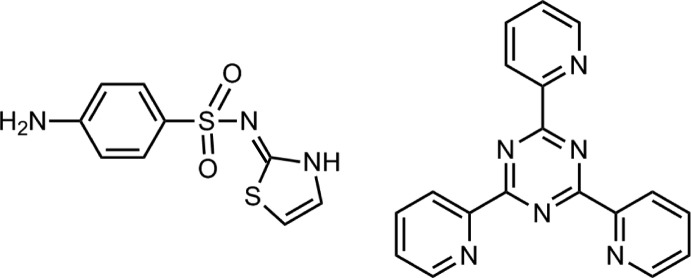


## Experimental   

### 

#### Crystal data  

C_18_H_12_N_6_·C_9_H_9_N_3_O_2_S_2_
*M* *_r_* = 567.65
Triclinic, 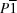

*a* = 8.8109 (13) Å
*b* = 12.7222 (16) Å
*c* = 13.1696 (14) Å
α = 66.227 (6)°
β = 73.797 (6)°
γ = 88.068 (9)°
*V* = 1292.1 (3) Å^3^
*Z* = 2
Mo *K*α radiation
μ = 0.25 mm^−1^
*T* = 98 K
0.49 × 0.45 × 0.05 mm

#### Data collection  

Rigaku AFC12/SATURN724 diffractometer
Absorption correction: multi-scan (*ABSCOR*; Higashi, 1995 ▶) *T*_min_ = 0.723, *T*_max_ = 1.000
8512 measured reflections
5885 independent reflections
5490 reflections with *I* > 2σ(*I*)
*R*_int_ = 0.022

#### Refinement  

*R*[*F* ^2^ > 2σ(*F* ^2^)] = 0.044
*wR*(*F* ^2^) = 0.087
*S* = 0.99
5885 reflections
371 parameters
H atoms treated by a mixture of independent and constrained refinement
Δρ_max_ = 0.48 e Å^−3^
Δρ_min_ = −0.39 e Å^−3^

### 

Data collection: *CrystalClear* (Molecular Structure Corporation & Rigaku, 2005 ▶); cell refinement: *CrystalClear*; data reduction: *CrystalClear*; program(s) used to solve structure: *SHELXS97* (Sheldrick, 2008 ▶); program(s) used to refine structure: *SHELXL97* (Sheldrick, 2008 ▶); molecular graphics: *ORTEP-3 for Windows* (Farrugia, 2012 ▶) and *DIAMOND* (Brandenburg, 2006 ▶); software used to prepare material for publication: *publCIF* (Westrip, 2010 ▶).

## Supplementary Material

Crystal structure: contains datablock(s) general, I. DOI: 10.1107/S1600536814004838/hg5388sup1.cif

Structure factors: contains datablock(s) I. DOI: 10.1107/S1600536814004838/hg5388Isup2.hkl

Supporting information file. DOI: 10.1107/S1600536814004838/hg5388Isup3.cml

CCDC reference: 989538

Additional supporting information:  crystallographic information; 3D view; checkCIF report

## Figures and Tables

**Table 1:** Hydrogen-bond geometry (Å, °)

*D*—H⋯*A*	*D*—H	H⋯*A*	*D*⋯*A*	*D*—H⋯*A*
N1—H1*N*⋯N9^i^	0.90 (3)	1.98 (3)	2.835 (3)	158 (2)
N3—H2*N*⋯N8^ii^	0.85 (3)	2.13 (3)	2.983 (3)	174 (2)
N3—H3*N*⋯N7^ii^	0.89 (2)	2.13 (2)	3.010 (2)	171 (3)
C2—H2⋯O2^iii^	0.95	2.37	3.237 (3)	151
C16—H16⋯O2^iv^	0.95	2.50	3.331 (2)	145
C20—H20⋯O1^v^	0.95	2.48	3.156 (3)	128

Symmetry codes: (i) 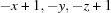; (ii) 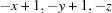; (iii) 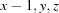; (iv) 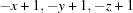; (v) 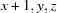.

**Fig. 1.**
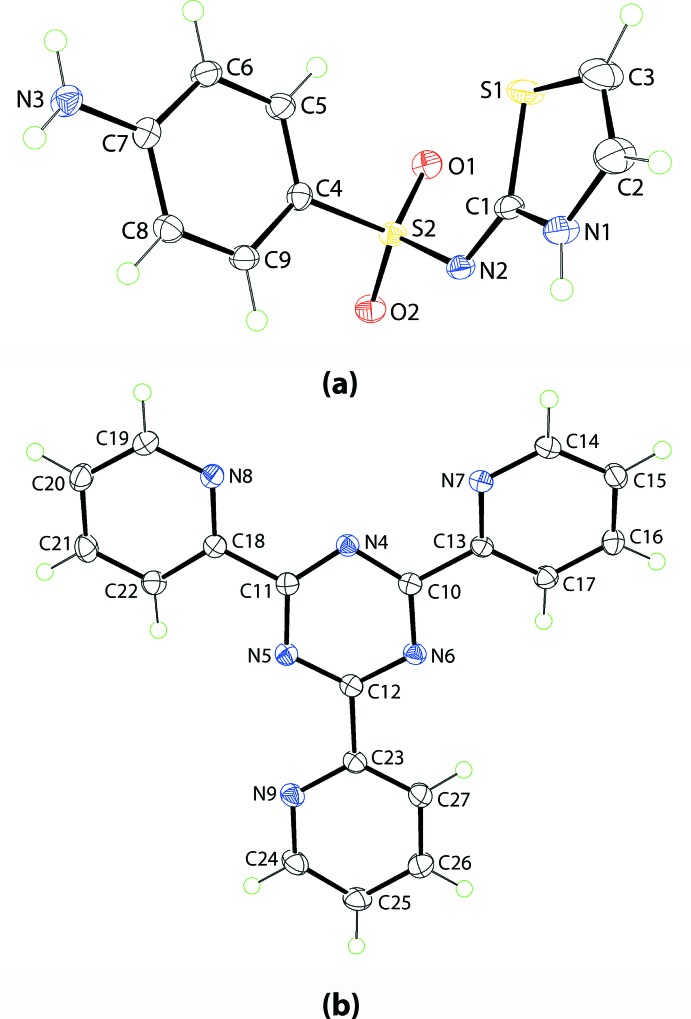
Molecular structures of (a) 4-amino-N-(1,3-thiazol-2(3H)-ylidene)benzenesulfonamide (sulfathiazole), and (b) 2,4,6-tris(pyridin-2-yl)-1,3,5-triazine, showing atom-labelling scheme and displacement ellipsoids at the 50% probability level.

**Fig. 2.**
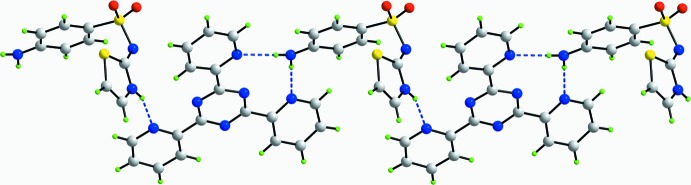
Supramolecular chains aligned along the [0 1 - 1] direction in (I) sustained by N—H···N hydrogen bonds which are shown as blue dashed lines.

**Fig. 3.**
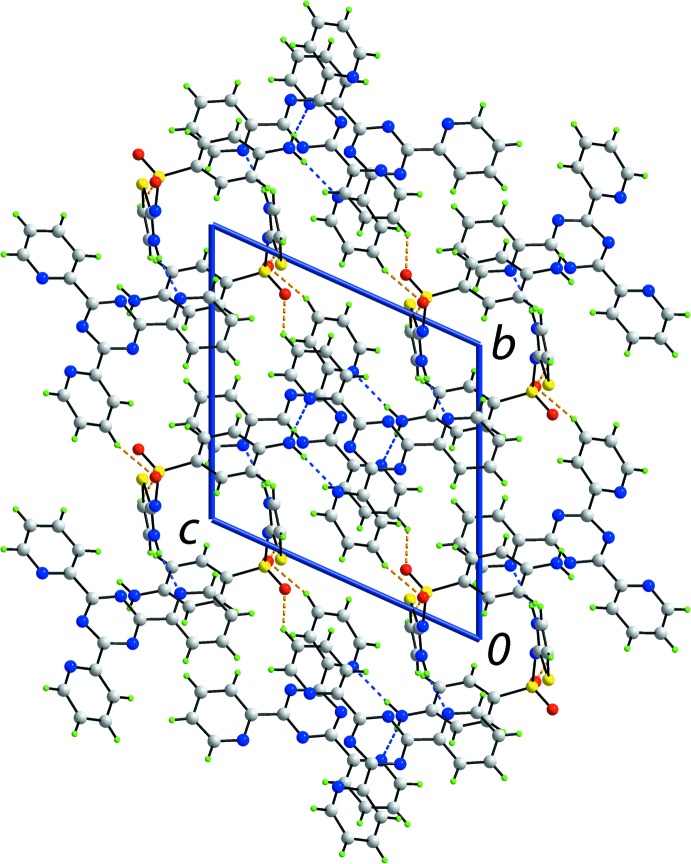
Unit-cell contents in (I) viewed in projection down the a axis. The N—H···N hydrogen bonds and C—H···O interactions are shown as blue and orange dashed lines, respectively.

## supplementary crystallographic information

### 2. Structural commentary

In continuation of co-crystallisation experiments involving sulfa­thia­zole (Arman *et al.*, 2012), herein, the crystal and molecular structure of the title co-crystal, (I), is described. Except for the description of polymorphic forms of the 1:1 co-crystals formed between sulfa­thia­zole and pyridine (Drebushchak *et al.*, 2006*a*; Drebushchak *et al.*, 2006*b*), no other reports of co-crystals of sulfa­thia­zole with pyridyl-containing molecules are known.

The components of co-crystal (I) are shown in Fig. 1. In the sulfa­thia­zole molecule, there is a twist about the S—N bond as seen in the value of the C4—S2—N2—C1 torsion angle of -77.54 (15)°. The dihedral angle between the five- and six-membered rings is 86.20 (9)°, so that the molecule has an overall *L*-shape. The observed conformation allows for the formation of an intra­molecular hypervalent S···O inter­action (O'Leary & Wallis, 2007), *i.e*. 2.8666 (15) Å. In the triazine molecule, the N7-, N8- and N9-containing pyridyl rings form dihedral angles of 18.35 (9), 6.12 (9) and 4.67 (9)°, respectively, with the central ring, indicating that overall the molecule has a disk shape. In terms of crystal packing (see below), crucially, the N7 and N8 atoms are directed toward each other, which facilitates the formation of amino-N—H···N(pyridyl) hydrogen bonding.

Table 1 summarises key hydrogen bonding contacts and Fig. 2 shows the association between the components of the co-crystal *via* amine-N—H with the N9-pydridyl ring of one triazine molecule, and between the amino-N—H atoms and the *syn*-disposed N7- and N8-pyridyl rings of another molecule, with the result that a linear supra­molecular chain is formed along [0 1 -1]. Chains are connected into a three-dimensional architecture by C—H···O inter­actions, Fig. 3 and Table 1.

### 5. Synthesis and crystallization

Sulfa­thia­zole (Sigma-Aldrich) and 2,4,6-tris­(pyridin-2-yl)-1,3,5-triazine (Sigma-Aldrich) were used as delivered. Single crystals of (I) used in the present study were harvested from a 1:1 acetone/ethanol (10 ml) solution of a 1:3 ratio of 2,4,6-tris­(pyridin-2-yl)-1,3,5-triazine (19 mg) and sulfa­thia­zole (46 mg) by slow evaporation of the solvent. M.pt: 471–475 K.

### 6. Refinement

C-bound H-atoms were placed in calculated positions (C—H 0.95 Å) and were included in the refinement in the riding model approximation with *U*_iso_(H) set to 1.2*U*_eq_(C). The N—H H-atoms were located in a difference Fourier map and refined without restraint for amine-H1n but with *U*_iso_(H) = 1.2*U*_eq_(N) in the cases of amino-H2n and H3n.

### Crystal data

**Table:**

C_18_H_12_N_6_·C_9_H_9_N_3_O_2_S_2_	*Z* = 2
*M**_r_* = 567.65	*F*(000) = 588
Triclinic, *P*1	*D*_x_ = 1.459 Mg m^−^^3^
Hall symbol: -P 1	Mo *K*α radiation, λ = 0.71073 Å
*a* = 8.8109 (13) Å	Cell parameters from 4931 reflections
*b* = 12.7222 (16) Å	θ = 3.0–40.2°
*c* = 13.1696 (14) Å	µ = 0.25 mm^−^^1^
α = 66.227 (6)°	*T* = 98 K
β = 73.797 (6)°	Platelet, gold
γ = 88.068 (9)°	0.49 × 0.45 × 0.05 mm
*V* = 1292.1 (3) Å^3^	

### Data collection

**Table:**

Rigaku AFC12K/SATURN724 diffractometer	5885 independent reflections
Radiation source: sealed tube	5490 reflections with *I* > 2σ(*I*)
Graphite monochromator	*R*_int_ = 0.022
Detector resolution: 28.5714 pixels mm^-1^	θ_max_ = 27.5°, θ_min_ = 2.4°
ω scans	*h* = −11→7
Absorption correction: multi-scan (*ABSCOR*; Higashi, 1995)	*k* = −16→12
*T*_min_ = 0.723, *T*_max_ = 1.000	*l* = −17→17
8512 measured reflections	

### Refinement

**Table:**

Refinement on *F*^2^	Primary atom site location: structure-invariant direct methods
Least-squares matrix: full	Secondary atom site location: difference Fourier map
*R*[*F*^2^ > 2σ(*F*^2^)] = 0.044	Hydrogen site location: inferred from neighbouring sites
*wR*(*F*^2^) = 0.087	H atoms treated by a mixture of independent and constrained refinement
*S* = 0.99	*w* = 1/[σ^2^(*F*_o_^2^) + (0.005*P*)^2^ + 1.820*P*] where *P* = (*F*_o_^2^ + 2*F*_c_^2^)/3
5885 reflections	(Δ/σ)_max_ < 0.001
371 parameters	Δρ_max_ = 0.48 e Å^−^^3^
0 restraints	Δρ_min_ = −0.39 e Å^−^^3^

### Special details

**Table:**

Geometry. All s.u.'s (except the s.u. in the dihedral angle between two l.s. planes) are estimated using the full covariance matrix. The cell s.u.'s are taken into account individually in the estimation of s.u.'s in distances, angles and torsion angles; correlations between s.u.'s in cell parameters are only used when they are defined by crystal symmetry. An approximate (isotropic) treatment of cell s.u.'s is used for estimating s.u.'s involving l.s. planes.
Refinement. Refinement of *F*^2^ against ALL reflections. The weighted *R*-factor *wR* and goodness of fit *S* are based on *F*^2^, conventional *R*-factors *R* are based on *F*, with *F* set to zero for negative *F*^2^. The threshold expression of *F*^2^ > 2σ(*F*^2^) is used only for calculating *R*-factors(gt) *etc*. and is not relevant to the choice of reflections for refinement. *R*-factors based on *F*^2^ are statistically about twice as large as those based on *F*, and *R*- factors based on ALL data will be even larger.

### Fractional atomic coordinates and isotropic or equivalent isotropic displacement parameters (Å^2^)

**Table:**

	*x*	*y*	*z*	*U*_iso_*/*U*_eq_	
S1	−0.04089 (5)	0.03018 (4)	0.25889 (5)	0.02706 (12)	
S2	0.34188 (5)	0.07956 (3)	0.19769 (3)	0.01620 (10)	
O1	0.23839 (15)	0.12511 (11)	0.27391 (10)	0.0209 (3)	
O2	0.49844 (15)	0.05721 (11)	0.21024 (11)	0.0223 (3)	
N1	0.04768 (19)	−0.14266 (13)	0.21606 (14)	0.0216 (3)	
H1N	0.105 (3)	−0.195 (2)	0.196 (2)	0.042 (7)*	
N2	0.26444 (17)	−0.04058 (12)	0.21139 (12)	0.0171 (3)	
N3	0.3779 (2)	0.37727 (13)	−0.28932 (13)	0.0206 (3)	
H2N	0.311 (3)	0.4266 (19)	−0.3067 (18)	0.025*	
H3N	0.424 (3)	0.3527 (18)	−0.3439 (19)	0.025*	
C1	0.1113 (2)	−0.05352 (14)	0.22511 (14)	0.0174 (3)	
C2	−0.1811 (2)	−0.06008 (18)	0.2549 (2)	0.0373 (5)	
H2	−0.2907	−0.0488	0.2670	0.045*	
C3	−0.1146 (2)	−0.14667 (18)	0.2330 (2)	0.0322 (5)	
H3	−0.1729	−0.2058	0.2292	0.039*	
C4	0.3594 (2)	0.17515 (14)	0.05455 (14)	0.0166 (3)	
C5	0.2596 (2)	0.26325 (15)	0.02888 (15)	0.0197 (3)	
H5	0.1867	0.2768	0.0898	0.024*	
C6	0.2662 (2)	0.33105 (15)	−0.08495 (15)	0.0203 (3)	
H6	0.1987	0.3917	−0.1014	0.024*	
C7	0.3712 (2)	0.31199 (14)	−0.17696 (14)	0.0168 (3)	
C8	0.4718 (2)	0.22258 (15)	−0.14895 (15)	0.0188 (3)	
H8	0.5445	0.2081	−0.2093	0.023*	
C9	0.4662 (2)	0.15621 (14)	−0.03585 (15)	0.0182 (3)	
H9	0.5355	0.0968	−0.0189	0.022*	
N4	0.71021 (17)	0.54364 (12)	0.51665 (12)	0.0169 (3)	
N5	0.80820 (17)	0.39937 (12)	0.65988 (12)	0.0178 (3)	
N6	0.65974 (18)	0.54222 (12)	0.70455 (12)	0.0180 (3)	
N7	0.50169 (17)	0.71447 (12)	0.47243 (12)	0.0178 (3)	
N8	0.83810 (18)	0.44100 (13)	0.36693 (13)	0.0202 (3)	
N9	0.82618 (18)	0.29923 (13)	0.88291 (13)	0.0209 (3)	
C10	0.6468 (2)	0.58500 (14)	0.59665 (14)	0.0169 (3)	
C11	0.7905 (2)	0.45104 (14)	0.55329 (14)	0.0171 (3)	
C12	0.7410 (2)	0.44915 (14)	0.73159 (14)	0.0173 (3)	
C13	0.5545 (2)	0.68775 (14)	0.56574 (14)	0.0164 (3)	
C14	0.4165 (2)	0.80561 (15)	0.44615 (15)	0.0195 (3)	
H14	0.3777	0.8251	0.3809	0.023*	
C15	0.3814 (2)	0.87340 (15)	0.50870 (15)	0.0211 (4)	
H15	0.3192	0.9367	0.4873	0.025*	
C16	0.4393 (2)	0.84643 (15)	0.60282 (16)	0.0225 (4)	
H16	0.4197	0.8921	0.6464	0.027*	
C17	0.5265 (2)	0.75155 (15)	0.63252 (15)	0.0200 (3)	
H17	0.5665	0.7305	0.6974	0.024*	
C18	0.8671 (2)	0.39881 (14)	0.47031 (15)	0.0175 (3)	
C19	0.9006 (2)	0.39032 (16)	0.29539 (16)	0.0236 (4)	
H19	0.8803	0.4190	0.2222	0.028*	
C20	0.9932 (2)	0.29850 (16)	0.32140 (16)	0.0242 (4)	
H20	1.0335	0.2646	0.2679	0.029*	
C21	1.0255 (2)	0.25734 (16)	0.42662 (16)	0.0236 (4)	
H21	1.0901	0.1952	0.4467	0.028*	
C22	0.9619 (2)	0.30844 (15)	0.50265 (15)	0.0205 (3)	
H22	0.9828	0.2822	0.5755	0.025*	
C23	0.7547 (2)	0.39769 (14)	0.85213 (15)	0.0185 (3)	
C24	0.8363 (2)	0.25076 (16)	0.99140 (16)	0.0253 (4)	
H24	0.8869	0.1813	1.0139	0.030*	
C25	0.7773 (2)	0.29598 (17)	1.07283 (16)	0.0262 (4)	
H25	0.7867	0.2582	1.1492	0.031*	
C26	0.7044 (2)	0.39730 (17)	1.04045 (16)	0.0274 (4)	
H26	0.6625	0.4306	1.0942	0.033*	
C27	0.6937 (2)	0.44959 (16)	0.92786 (16)	0.0238 (4)	
H27	0.6453	0.5198	0.9031	0.029*	

### Atomic displacement parameters (Å^2^)

**Table:**

	*U*^11^	*U*^22^	*U*^33^	*U*^12^	*U*^13^	*U*^23^
S1	0.0168 (2)	0.0232 (2)	0.0442 (3)	0.00686 (17)	−0.0085 (2)	−0.0174 (2)
S2	0.0161 (2)	0.0180 (2)	0.01595 (19)	0.00379 (15)	−0.00736 (15)	−0.00686 (15)
O1	0.0245 (7)	0.0229 (6)	0.0183 (6)	0.0043 (5)	−0.0080 (5)	−0.0105 (5)
O2	0.0174 (6)	0.0265 (7)	0.0237 (6)	0.0041 (5)	−0.0113 (5)	−0.0078 (5)
N1	0.0203 (8)	0.0193 (7)	0.0298 (8)	0.0049 (6)	−0.0112 (6)	−0.0123 (6)
N2	0.0167 (7)	0.0161 (7)	0.0189 (7)	0.0044 (5)	−0.0068 (6)	−0.0067 (6)
N3	0.0255 (8)	0.0205 (7)	0.0166 (7)	0.0049 (6)	−0.0076 (6)	−0.0076 (6)
C1	0.0180 (8)	0.0169 (8)	0.0175 (8)	0.0059 (6)	−0.0072 (6)	−0.0062 (6)
C2	0.0188 (10)	0.0312 (11)	0.0659 (16)	0.0044 (8)	−0.0167 (10)	−0.0212 (11)
C3	0.0242 (10)	0.0269 (10)	0.0508 (13)	0.0019 (8)	−0.0187 (9)	−0.0162 (9)
C4	0.0176 (8)	0.0171 (8)	0.0147 (7)	0.0005 (6)	−0.0055 (6)	−0.0055 (6)
C5	0.0220 (9)	0.0200 (8)	0.0174 (8)	0.0059 (7)	−0.0049 (7)	−0.0090 (7)
C6	0.0226 (9)	0.0180 (8)	0.0207 (8)	0.0069 (7)	−0.0074 (7)	−0.0078 (7)
C7	0.0183 (8)	0.0160 (8)	0.0175 (8)	−0.0017 (6)	−0.0071 (6)	−0.0066 (6)
C8	0.0177 (8)	0.0212 (8)	0.0184 (8)	0.0035 (6)	−0.0046 (7)	−0.0095 (7)
C9	0.0159 (8)	0.0179 (8)	0.0215 (8)	0.0044 (6)	−0.0065 (7)	−0.0081 (7)
N4	0.0177 (7)	0.0169 (7)	0.0168 (7)	0.0015 (5)	−0.0063 (6)	−0.0068 (5)
N5	0.0177 (7)	0.0179 (7)	0.0187 (7)	0.0018 (5)	−0.0068 (6)	−0.0076 (6)
N6	0.0214 (7)	0.0164 (7)	0.0174 (7)	0.0022 (6)	−0.0080 (6)	−0.0067 (6)
N7	0.0191 (7)	0.0172 (7)	0.0163 (7)	0.0018 (5)	−0.0060 (6)	−0.0054 (6)
N8	0.0216 (8)	0.0211 (7)	0.0193 (7)	0.0038 (6)	−0.0065 (6)	−0.0096 (6)
N9	0.0245 (8)	0.0197 (7)	0.0210 (7)	0.0046 (6)	−0.0115 (6)	−0.0079 (6)
C10	0.0170 (8)	0.0166 (8)	0.0173 (8)	−0.0002 (6)	−0.0062 (6)	−0.0062 (6)
C11	0.0162 (8)	0.0165 (8)	0.0180 (8)	−0.0007 (6)	−0.0046 (6)	−0.0067 (6)
C12	0.0181 (8)	0.0161 (8)	0.0185 (8)	−0.0001 (6)	−0.0073 (7)	−0.0065 (6)
C13	0.0163 (8)	0.0155 (8)	0.0159 (7)	0.0005 (6)	−0.0044 (6)	−0.0048 (6)
C14	0.0192 (8)	0.0198 (8)	0.0179 (8)	0.0018 (6)	−0.0071 (7)	−0.0051 (7)
C15	0.0204 (9)	0.0181 (8)	0.0225 (8)	0.0047 (7)	−0.0059 (7)	−0.0064 (7)
C16	0.0271 (9)	0.0187 (8)	0.0229 (9)	0.0040 (7)	−0.0060 (7)	−0.0108 (7)
C17	0.0236 (9)	0.0192 (8)	0.0185 (8)	0.0017 (7)	−0.0076 (7)	−0.0080 (7)
C18	0.0163 (8)	0.0170 (8)	0.0183 (8)	−0.0009 (6)	−0.0039 (6)	−0.0068 (6)
C19	0.0278 (10)	0.0259 (9)	0.0193 (8)	0.0041 (7)	−0.0075 (7)	−0.0111 (7)
C20	0.0241 (9)	0.0252 (9)	0.0238 (9)	0.0038 (7)	−0.0028 (7)	−0.0133 (7)
C21	0.0192 (9)	0.0227 (9)	0.0281 (9)	0.0063 (7)	−0.0052 (7)	−0.0109 (7)
C22	0.0183 (8)	0.0219 (8)	0.0205 (8)	0.0026 (7)	−0.0057 (7)	−0.0079 (7)
C23	0.0197 (8)	0.0176 (8)	0.0192 (8)	0.0011 (6)	−0.0082 (7)	−0.0067 (7)
C24	0.0308 (10)	0.0219 (9)	0.0252 (9)	0.0067 (7)	−0.0154 (8)	−0.0071 (7)
C25	0.0322 (10)	0.0284 (10)	0.0195 (9)	0.0049 (8)	−0.0141 (8)	−0.0073 (7)
C26	0.0342 (11)	0.0299 (10)	0.0225 (9)	0.0077 (8)	−0.0115 (8)	−0.0134 (8)
C27	0.0290 (10)	0.0229 (9)	0.0224 (9)	0.0080 (7)	−0.0113 (8)	−0.0098 (7)

### Geometric parameters (Å, º)

**Table:**

S1—C2	1.739 (2)	N7—C13	1.347 (2)
S1—C1	1.7559 (17)	N8—C19	1.335 (2)
S2—O2	1.4401 (13)	N8—C18	1.343 (2)
S2—O1	1.4482 (13)	N9—C24	1.337 (2)
S2—N2	1.6167 (15)	N9—C23	1.345 (2)
S2—C4	1.7489 (17)	C10—C13	1.490 (2)
N1—C1	1.343 (2)	C11—C18	1.495 (2)
N1—C3	1.384 (2)	C12—C23	1.492 (2)
N1—H1N	0.90 (3)	C13—C17	1.391 (2)
N2—C1	1.317 (2)	C14—C15	1.390 (2)
N3—C7	1.359 (2)	C14—H14	0.9500
N3—H2N	0.86 (2)	C15—C16	1.383 (3)
N3—H3N	0.89 (2)	C15—H15	0.9500
C2—C3	1.326 (3)	C16—C17	1.388 (2)
C2—H2	0.9500	C16—H16	0.9500
C3—H3	0.9500	C17—H17	0.9500
C4—C5	1.392 (2)	C18—C22	1.393 (2)
C4—C9	1.397 (2)	C19—C20	1.385 (3)
C5—C6	1.381 (2)	C19—H19	0.9500
C5—H5	0.9500	C20—C21	1.379 (3)
C6—C7	1.407 (2)	C20—H20	0.9500
C6—H6	0.9500	C21—C22	1.388 (2)
C7—C8	1.411 (2)	C21—H21	0.9500
C8—C9	1.372 (2)	C22—H22	0.9500
C8—H8	0.9500	C23—C27	1.388 (2)
C9—H9	0.9500	C24—C25	1.385 (3)
N4—C11	1.341 (2)	C24—H24	0.9500
N4—C10	1.341 (2)	C25—C26	1.382 (3)
N5—C12	1.337 (2)	C25—H25	0.9500
N5—C11	1.341 (2)	C26—C27	1.389 (3)
N6—C12	1.337 (2)	C26—H26	0.9500
N6—C10	1.338 (2)	C27—H27	0.9500
N7—C14	1.338 (2)		
			
C2—S1—C1	91.05 (9)	N4—C11—C18	118.45 (15)
O2—S2—O1	117.20 (8)	N5—C11—C18	116.06 (15)
O2—S2—N2	105.59 (8)	N6—C12—N5	125.13 (15)
O1—S2—N2	111.61 (8)	N6—C12—C23	116.30 (15)
O2—S2—C4	108.79 (8)	N5—C12—C23	118.56 (15)
O1—S2—C4	108.12 (8)	N7—C13—C17	122.90 (16)
N2—S2—C4	104.82 (8)	N7—C13—C10	117.34 (15)
C1—N1—C3	115.58 (16)	C17—C13—C10	119.76 (15)
C1—N1—H1N	123.4 (16)	N7—C14—C15	123.83 (16)
C3—N1—H1N	120.9 (16)	N7—C14—H14	118.1
C1—N2—S2	119.62 (12)	C15—C14—H14	118.1
C7—N3—H2N	121.0 (14)	C16—C15—C14	118.37 (16)
C7—N3—H3N	120.2 (14)	C16—C15—H15	120.8
H2N—N3—H3N	115 (2)	C14—C15—H15	120.8
N2—C1—N1	121.27 (15)	C15—C16—C17	118.85 (16)
N2—C1—S1	129.99 (13)	C15—C16—H16	120.6
N1—C1—S1	108.72 (13)	C17—C16—H16	120.6
C3—C2—S1	111.04 (16)	C16—C17—C13	118.88 (16)
C3—C2—H2	124.5	C16—C17—H17	120.6
S1—C2—H2	124.5	C13—C17—H17	120.6
C2—C3—N1	113.58 (18)	N8—C18—C22	122.68 (16)
C2—C3—H3	123.2	N8—C18—C11	117.51 (15)
N1—C3—H3	123.2	C22—C18—C11	119.80 (15)
C5—C4—C9	119.38 (15)	N8—C19—C20	123.98 (17)
C5—C4—S2	121.31 (13)	N8—C19—H19	118.0
C9—C4—S2	119.07 (13)	C20—C19—H19	118.0
C6—C5—C4	120.18 (16)	C21—C20—C19	118.51 (17)
C6—C5—H5	119.9	C21—C20—H20	120.7
C4—C5—H5	119.9	C19—C20—H20	120.7
C5—C6—C7	121.20 (16)	C20—C21—C22	118.67 (17)
C5—C6—H6	119.4	C20—C21—H21	120.7
C7—C6—H6	119.4	C22—C21—H21	120.7
N3—C7—C6	121.83 (16)	C21—C22—C18	118.91 (17)
N3—C7—C8	120.52 (16)	C21—C22—H22	120.5
C6—C7—C8	117.64 (15)	C18—C22—H22	120.5
C9—C8—C7	121.02 (16)	N9—C23—C27	122.59 (16)
C9—C8—H8	119.5	N9—C23—C12	116.79 (15)
C7—C8—H8	119.5	C27—C23—C12	120.61 (16)
C8—C9—C4	120.57 (16)	N9—C24—C25	123.73 (17)
C8—C9—H9	119.7	N9—C24—H24	118.1
C4—C9—H9	119.7	C25—C24—H24	118.1
C11—N4—C10	114.23 (14)	C26—C25—C24	118.44 (17)
C12—N5—C11	114.75 (15)	C26—C25—H25	120.8
C12—N6—C10	114.91 (15)	C24—C25—H25	120.8
C14—N7—C13	117.16 (15)	C25—C26—C27	118.78 (18)
C19—N8—C18	117.21 (16)	C25—C26—H26	120.6
C24—N9—C23	117.49 (16)	C27—C26—H26	120.6
N6—C10—N4	125.46 (16)	C23—C27—C26	118.97 (17)
N6—C10—C13	115.56 (15)	C23—C27—H27	120.5
N4—C10—C13	118.97 (15)	C26—C27—H27	120.5
N4—C11—N5	125.49 (15)		
			
O2—S2—N2—C1	167.64 (13)	C11—N5—C12—N6	0.7 (2)
O1—S2—N2—C1	39.26 (16)	C11—N5—C12—C23	−179.92 (15)
C4—S2—N2—C1	−77.54 (15)	C14—N7—C13—C17	−1.2 (2)
S2—N2—C1—N1	167.95 (13)	C14—N7—C13—C10	178.86 (15)
S2—N2—C1—S1	−13.5 (2)	N6—C10—C13—N7	−162.39 (15)
C3—N1—C1—N2	179.67 (17)	N4—C10—C13—N7	18.1 (2)
C3—N1—C1—S1	0.9 (2)	N6—C10—C13—C17	17.7 (2)
C2—S1—C1—N2	179.95 (18)	N4—C10—C13—C17	−161.75 (16)
C2—S1—C1—N1	−1.38 (15)	C13—N7—C14—C15	0.5 (3)
C1—S1—C2—C3	1.62 (19)	N7—C14—C15—C16	0.9 (3)
S1—C2—C3—N1	−1.4 (3)	C14—C15—C16—C17	−1.5 (3)
C1—N1—C3—C2	0.4 (3)	C15—C16—C17—C13	0.8 (3)
O2—S2—C4—C5	−140.37 (14)	N7—C13—C17—C16	0.6 (3)
O1—S2—C4—C5	−12.10 (17)	C10—C13—C17—C16	−179.51 (16)
N2—S2—C4—C5	107.07 (15)	C19—N8—C18—C22	2.0 (3)
O2—S2—C4—C9	45.36 (16)	C19—N8—C18—C11	−176.87 (16)
O1—S2—C4—C9	173.63 (13)	N4—C11—C18—N8	−6.3 (2)
N2—S2—C4—C9	−67.20 (15)	N5—C11—C18—N8	173.32 (15)
C9—C4—C5—C6	0.1 (3)	N4—C11—C18—C22	174.83 (16)
S2—C4—C5—C6	−174.18 (14)	N5—C11—C18—C22	−5.6 (2)
C4—C5—C6—C7	1.0 (3)	C18—N8—C19—C20	−0.5 (3)
C5—C6—C7—N3	179.31 (17)	N8—C19—C20—C21	−1.0 (3)
C5—C6—C7—C8	−1.2 (3)	C19—C20—C21—C22	1.0 (3)
N3—C7—C8—C9	179.96 (16)	C20—C21—C22—C18	0.4 (3)
C6—C7—C8—C9	0.5 (3)	N8—C18—C22—C21	−2.0 (3)
C7—C8—C9—C4	0.5 (3)	C11—C18—C22—C21	176.84 (16)
C5—C4—C9—C8	−0.8 (3)	C24—N9—C23—C27	0.5 (3)
S2—C4—C9—C8	173.57 (14)	C24—N9—C23—C12	−178.59 (16)
C12—N6—C10—N4	−1.4 (2)	N6—C12—C23—N9	175.31 (15)
C12—N6—C10—C13	179.14 (14)	N5—C12—C23—N9	−4.1 (2)
C11—N4—C10—N6	0.7 (2)	N6—C12—C23—C27	−3.8 (2)
C11—N4—C10—C13	−179.88 (14)	N5—C12—C23—C27	176.81 (17)
C10—N4—C11—N5	0.9 (2)	C23—N9—C24—C25	0.2 (3)
C10—N4—C11—C18	−179.52 (15)	N9—C24—C25—C26	−0.4 (3)
C12—N5—C11—N4	−1.6 (2)	C24—C25—C26—C27	−0.1 (3)
C12—N5—C11—C18	178.84 (14)	N9—C23—C27—C26	−1.0 (3)
C10—N6—C12—N5	0.7 (2)	C12—C23—C27—C26	178.08 (17)
C10—N6—C12—C23	−178.71 (15)	C25—C26—C27—C23	0.7 (3)

### Hydrogen-bond geometry (Å, º)

**Table:**

*D*—H···*A*	*D*—H	H···*A*	*D*···*A*	*D*—H···*A*
N1—H1*N*···N9^i^	0.90 (3)	1.98 (3)	2.835 (3)	158 (2)
N3—H2*N*···N8^ii^	0.85 (3)	2.13 (3)	2.983 (3)	174 (2)
N3—H3*N*···N7^ii^	0.89 (2)	2.13 (2)	3.010 (2)	171 (3)
C2—H2···O2^iii^	0.95	2.37	3.237 (3)	151
C16—H16···O2^iv^	0.95	2.50	3.331 (2)	145
C20—H20···O1^v^	0.95	2.48	3.156 (3)	128

Symmetry codes: (i) −*x*+1, −*y*, −*z*+1; (ii) −*x*+1, −*y*+1, −*z*; (iii) *x*−1, *y*, *z*; (iv) −*x*+1, −*y*+1, −*z*+1; (v) *x*+1, *y*, *z*.
